# Resources to Support Decision-Making Regarding End-of-Life Nutrition Care in Long-Term Care: A Scoping Review

**DOI:** 10.3390/nu16081163

**Published:** 2024-04-13

**Authors:** Heather Alford, Nadia Anvari, Christina Lengyel, Abigail Wickson-Griffiths, Paulette Hunter, Erin Yakiwchuk, Allison Cammer

**Affiliations:** 1 College of Pharmacy and Nutrition, University of Saskatchewan, Saskatoon, SK S7N 5E5, Canada; heather.alford@usask.ca (H.A.); nadia.anvari@usask.ca (N.A.); emk092@mail.usask.ca (E.Y.); 2 Faculty of Agricultural and Food Sciences, University of Manitoba, Winnipeg, MB R3T 2N2, Canada; christina.lengyel@umanitoba.ca; 3 Faculty of Nursing, University of Regina, Regina, SK S4S 0A2, Canada; abigail.wickson-griffiths@uregina.ca; 4 St. Thomas More College, University of Saskatchewan, Saskatoon, SK S7N 0W6, Canada; paulette.hunter@usask.ca

**Keywords:** end of life, nutrition care, artificial nutrition and hydration, long-term care

## Abstract

Resources are needed to aid healthcare providers and families in making end-of-life nutrition care decisions for residents living in long-term care settings. This scoping review aimed to explore what is reported in the literature about resources to support decision-making at the end of life in long-term care. Four databases were searched for research published from 2003 to June 2023. Articles included peer-reviewed human studies published in the English language that reported resources to support decision-making about end-of-life nutrition in long-term care settings. In total, 15 articles were included. Thematic analysis of the articles generated five themes: conversations about care, evidence-based decision-making, a need for multidisciplinary perspectives, honouring residents’ goals of care, and cultural considerations for adapting resources. Multidisciplinary care teams supporting residents and their families during the end of life can benefit from resources to support discussion and facilitate decision-making.

## 1. Introduction

End of life (EOL), defined as the terminal phase of life, often represents a stressful and challenging time for long-term care (LTC) residents and their families [1,2,3]. With an estimated 25–30% of LTC residents dying annually, quality EOL care is essential [4,5]. The goals of nutrition care in LTC are restoration/rehabilitation or maintenance and preservation of function, but at EOL, the focus is solely on quality of life and quality of care, emphasizing the symbolic and pleasurable qualities of eating and drinking [1,2]. This can be an abrupt shift in the goals of care that can be challenging for both families and healthcare providers (HCPs) to embrace [6].

The medical management of EOL is complex. In terms of nutrition care, there is no consensus diagnosis or prognostication that enables the confident assertion that it is the right time to change the goals of nutrition care to terminal care, focusing solely on comfort measures [1,7]. In fact, clinical scales often include eating and drinking as sentinel acts that signal EOL (e.g., the Palliative Performance Scale [8]), and disinterest in eating and drinking can be confounded as a sign of depression or apathy. Instead, HCPs monitor clinical signs and work with residents and families to make complex decisions about nutrition at EOL [9]. With a high prevalence of dementia in LTC and a high proportion of LTC residents rated as cognitively impaired [10], capacity can be limited, and decision-making about nutrition care falls to substitute decision-makers (often family members), which can be very stressful [3,6,7,11,12,13]. Both family members and HCPs experience moral strain about EOL nutrition, questioning whether to pursue aggressive nutrition care or discontinue it [14,15,16,17].

Within LTC, eating challenges are very common [18]. While much attention is focused on strategies to mitigate these challenges and increase intake, attention is needed on supporting declining intake as a function of EOL. Eating and drinking often progressively decrease as residents approach EOL, but the trajectory is varied and often difficult to anticipate; some may maintain pleasure from intake and others may experience discomfort, fatigue, or pain from intake [1,6]. Often, artificial nutrition and hydration (ANH) interventions are sought (e.g., medical enteral/parenteral feeding; clinical hydration via intravenous, nasogastric, hypodermoclysis, rectal) despite little evidence to support use [1,7,17,19,20,21]. Both mechanical and physiological harms are known to result from aggressive ANH at EOL (e.g., discomfort, restriction in mobility, pulmonary edema, nausea, and vomiting), yet HCPs and families often request such interventions [1,13,21,22,23]. Enteral tube feeding persists among residents with dementia at EOL despite strong evidence against its use [24,25,26]. Fear persists that withdrawing nutrition therapy is painful despite known physiological processes that support the amelioration of pain when nutrition is discontinued at EOL (e.g., azotemia, hyponatremia) [1,23,27]. Criteria to discontinue ANH are lacking; families find withdrawing ANH particularly difficult [16,28,29], and performance status scales (e.g., ECOG Performance Status Scale [30], Karnofsky Performance Status Scale [31]) are not helpful for determining when to shift nutrition goals of care. In terms of the clinical management of EOL, decisions about artificial nutrition create more ethical dilemmas than other treatments, including artificial hydration and antibiotic therapy [17]. Yet, discussion about nutrition and hydration decisions is often not germane to care, and evidence suggests that HCPs do not consistently involve residents and families in these important healthcare decisions [9].

Once strategies to address eating and drinking challenges have been exhausted, supports for transitioning into EOL care are needed [7]. Often, this is framed as compassionate terminal care or ‘comfort feeding only’. In this approach, quality of life is emphasized, and nutritional adequacy and functional properties of eating are discarded [32]. In this approach, there is flexibility to respond to the resident’s abilities and desires without repeated clinical assessments to determine physiological safety (e.g., dysphagia) and changes in their nutritional care plan. This can occur before active dying begins (i.e., days or hours to live) and can support HCPs and families in understanding that the EOL stage of care has begun [32].

Previous literature suggests that families lack information about how to incorporate nutrition and hydration into care plans to optimize quality of life at the EOL [33,34]. Conversely, research has shown that families who had a single, in-depth conversation with HCPs about EOL care options for LTC home residents living with advanced dementia had significantly higher satisfaction with care and documented significantly more decisions in their family resident’s advance care plan [35]. Because of the medical, emotional, and ethical complexity of EOL nutrition care, there is a need for resources to support HCPs and families in having discussions and making decisions about EOL nutrition care. Defined as evidence-based resources to support participation in healthcare choices [36,37], decision aids provide information about a health condition and the benefits and risks of treatment options to supplement patient/family-provider conversations about care and support values-based decision-making about care [38]. The purpose of this study was to conduct a scoping review of resources to support EOL decision-making for nutrition and hydration in the LTC context. The research question guiding the study was: What is reported in the literature about resources to support decision-making about EOL nutrition care in LTC settings?

## 2. Methods

We followed Levac et al.’s [39] adaptations of the Arksey and O’Malley [40] Click or tap here to enter text.six-stage framework for conducting a scoping study: identifying the research question, identifying relevant studies, study selection, charting the data, collating, summarizing, and reporting results, and consultation. With recommendations for enhancing each stage of the framework, Levac et al. [39] sought to better position researchers to achieve sufficient detail and description of analysis processes to facilitate greater consistency in scoping review processes and reporting. Recommended enhancements that were followed for this scoping review include linking the purpose and research question, balancing feasibility with the breadth of the scoping process, selecting studies and extracting data using an iterative team approach, incorporating qualitative thematic analysis, identifying implications for policy, practice, or research, and undergoing consultation as part of the scoping study methodology [39].

### 2.1. Search Strategy

Search terms were developed through consultation with a university librarian and the Saskatchewan Long-Term Care Network, a group of patient-family partners, clinicians, and academics working together on initiatives to improve LTC in Saskatchewan, Canada. Feedback was obtained to ensure search terms encompassed all concepts relevant to the research question. Search terms are listed in Table 1.

We searched MEDLINE, CINAHL, Web of Science, and Embase. Preliminary searches were conducted to evaluate the research question, search terms, and scope of results. A search was conducted on 13 June 2023. A total of 1363 papers were retrieved and uploaded to Rayyan software [41] for de-duplication, after which 1038 remained. The use of the “blind option” in the Rayyan software [41] allowed independent review and selection of all records by the researchers. Two researchers (HA and NA) independently screened titles and abstracts against inclusion and exclusion criteria. Conflicts were resolved by a third reviewer (AC). The research articles included were peer-reviewed human studies published in the English language from 2003 to June 2023 that reported tangible resources to support decision-making about EOL nutrition in LTC. Articles were excluded if they were non-human studies, published in a non-English language, a review article, or published prior to 2003. After initial screening, 274 papers remained. Of the 274 articles, 71 were excluded due to not being specific to the LTC setting, 90 did not have specific content pertaining to nutrition, and 134 were not research papers (e.g., editorial, narrative description). Under the supervision of AC, researchers HA and NA conducted independent full-text screenings of the articles against the inclusion and exclusion criteria. The citation lists of the remaining articles were then hand-searched by NA and cross checked by HA to identify potential references that met the inclusion criteria. An additional 39 papers were selected and screened by HA and NA, of which five were included. This rendered a total of 18 papers that advanced for quality appraisal.

### 2.2. Quality Appraisal

**Figure 1:**
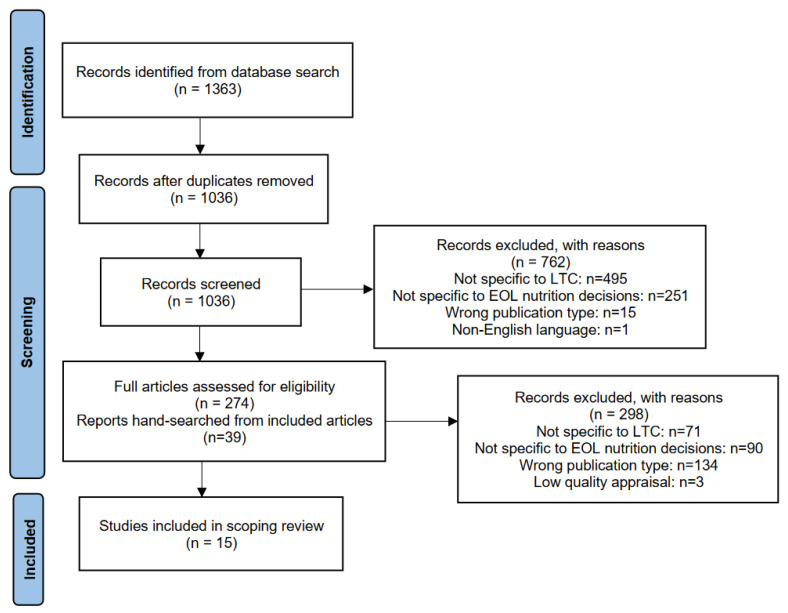
Chart of the search process.

Using the Critical Appraisal Skills Programme (CASP) checklists [42], the quality of the included articles was appraised independently by reviewers HA and NA and confirmed by AC. The CASP checklists provided an opportunity for discussion among the researchers about the quality and content of the studies. Though CASP checklists do not provide a cut-off score for low quality, three articles were excluded based on consensus agreement that they were of low quality and misfit with the research question. The search and article screening are presented in a chart of search results (Figure 1).

A total of 15 papers were included for analysis in the scoping review.

### 2.3. Data Analysis

The following data were summarized from the included studies: publication year, study location, design, key findings, and implications. Further, the following data were extracted to characterize the decision aids related to each study: type of decision aid, target audience, goals of the study, and existing gaps. These findings are summarized in table form (Table 2).

Following the extraction of the above data, thematic analysis was conducted by researchers HA and NA under the guidance of AC to identify key themes captured in the included studies.

## 3. Results

In total, 15 papers related to 12 different resources for supporting nutrition care decisions at EOL were included in this scoping review. All papers represented research undertaken in high-income countries. The resources span a variety of types and purposes, and the corresponding research approaches and findings vary accordingly. See Table 2 for an overview of included studies. Five key themes were identified from the studies: conversations about care, evidence-based decision-making, a need for multidisciplinary perspectives, honouring residents’ goals of care, and cultural considerations for adapting resources.

### 3.1. Conversations about Care

Each of the included studies emphasized the importance of conversations between residents, families, and HCPs for aligning goals of care, aiding in the shared decision-making process, and optimizing the quality of decisions made. Formal documents, such as advance directives or advance care plans, were recommended for promoting resident autonomy and involvement in treatment decisions [43,44,45,46,47,48,49,50,51,52,53,54,55,56,57]. Care conferences were identified as key opportunities for more formal conversations, and regular informal conversations were equally important opportunities for HCPs to communicate changes in a resident’s health and to clarify goals of care with residents and families [43]. For each resource/intervention, the authors identified the importance of early distribution and usage for promoting more frequent and in-depth conversations about care, particularly regarding the course of dementia and available treatment options to avoid unwanted, futile, or burdensome interventions [43,44,50]. Use of resources to support decision-making was consistently associated with more frequent discussions amongst families and HCPs [43,50], with greater impact in facilities with fewer nurse practitioners or physician assistants present [49], and greater family participation in decision-making [45,52]. Additionally, an international team of researchers found that such resources can be used to overcome staff-family communication barriers [57].

### 3.2. Evidence-Based Decision-Making

Some authors posited that because of a lack of information on the risks and marginal benefits of certain ANH interventions in EOL dementia care, such interventions may placate families or caregivers by providing the sense that efforts were exhausted to preserve nutritional intake [50,53]. In fact, tube feedings were perceived by staff and families alike as providing nourishment and care, despite the lack of evidence for a clear and consistent benefit to residents [50,53,55]. With the use of resources to support decision-making, carers had increased knowledge about treatments [46,50,51,53], significantly better decisional capacity [45], reduced decisional conflict [49,50,51,53] and ultimately were more likely to avoid unnecessary or burdensome interventions, including tube feeding [50,53]. Such resources prompted and provided frameworks for conversations and presented unequivocal information about treatments, which some equated to myth-busting, making it easier to arrive at a decision.

### 3.3. A Need for Multidisciplinary Perspectives

Care teams were best positioned to support residents and families when bolstered with multiple HCP perspectives. Specifically, nurse practitioners were named as a key human health resource whose proximity to the care team helped to inform and support both families and other HCPs, namely physicians [47,56]. Most authors reiterated that the importance of a cohesive and collaborative care team cannot be overemphasized. On the other hand, none of the studies mentioned the role of registered dietitians except for one that noted the lack of dietitian involvement as a limitation to the study [46]. Moreover, only two decision aids were developed in partnership with residents and families using a co-design approach [43,46]. These two resources focus on family satisfaction with EOL care. Many studies did, however, test the content, effectiveness, and acceptability of the respective resources among family [43,49,50,51,52,53,56] and resident populations, including residents living with dementia [45,50].

### 3.4. Honouring Residents’ Goals of Care

Of central importance within each resource was to respect and honour each resident’s goals of care. Most authors noted the importance of advance care plans for documenting residents’ preferences. In instances where residents’ wishes were not made clear (e.g., using an advance care plan) or a resident lacked capacity to share their preferences, authors advised that caregivers use their best judgment based on personal knowledge of the resident [44,52,55]. Resources to support decisions are typically presented and clarified for each aspect of care for families and HCPs to consider. Since enhancing a resident’s quality of life in accordance with their goals of care was of primary importance, many such resources provided synopses of treatment options, thus providing the information necessary to weigh the associated benefits, risks, and burdens of available options [45,46,50]. Specifically, providing families and HCPs with knowledge of the course of the illness trajectory towards EOL was important for understanding prognoses and minimizing further stress and anxiety related to observed changes in residents during EOL [55].

### 3.5. Cultural Considerations for Adapting Resources

One resource, a comfort care booklet [58], originally developed in Canada in English and French, was adapted for implementation in Italy, Japan, and the Netherlands [59]. Because of cultural differences in EOL care provision, an international team of researchers worked to adapt the booklet, revising the content to meet local legal and ethical frameworks. The translated versions of the booklet were evaluated for acceptability and usefulness among HCPs [44,48,57] and families [56]. While the need for and perceived usefulness of the booklet was nearly universal among HCPs and families [44,56], evaluations of its format varied for staff and families, denoting the importance of cultural context. One study found that resources to support EOL care were endorsed, particularly among nurses, regardless of format [59]. Families were more sensitive to the format of the booklet, with the authors suggesting greater adaptation to text and photos to better suit families’ preferences for obtaining information [56].

## 4. Discussion

Choices about eating and drinking are some of the most common yet challenging decisions that families and HCPs face in LTC [16,60]. This scoping review aimed to understand what resources exist to support decision-making about EOL nutrition care within the LTC setting. Decision aids provide structured, evidence-based information about the risks and benefits of available treatments, improving the quality and efficiency of decision-making for HCPs and families [46,50,52,61]. Other resources to support decision-making include descriptions of illness trajectories, information about treatment and care options, and advance care plans. Yet few tangible resources are available to aid decision-making about EOL nutrition in LTC. For those that have been reported in the literature, implementing such resources increased knowledge about EOL nutrition for HCPs and families [50,52,53], reduced decisional conflict [49,50,51,53], increased staff-family communication [43,49,50], and better positioned carers to consider available treatment options while prioritizing resident goals of care and quality of life.

There is a clear need for increased conversation and better support for families in EOL nutrition care decision-making [3,6,13,62]. Engaging families and HCPs in discussions about care facilitates shared and informed decision-making that leads to better health outcomes and quality of care [61]. While staff shortages and time constraints are commonly identified as barriers to staff-family discussions about care, clinical judgment does not supersede facilitating shared decision-making among care partners. For instance, Roach et al. [62] found that staff conflated caregiver guilt with desire for ANH, including tube feeding. The authors warned that in the absence of staff-family conversations, staff biases and assumptions may lead to more aggressive and burdensome treatments than are aligned with families’ actual goals or preferences. Similarly, other studies show that staff-family conflicts can arise when HCPs disregard patient/family wishes or pursue unwanted, invasive treatments, contributing to unease and distress or increasing caregiver burden for families [3,63]. Resources to support decision-making can be used to mitigate time constraints that limit opportunities for staff-family discussions by making the necessary information available to reach an informed consensus that optimizes the quality of decisions made and care provided.

Since the roles of most HCPs are not well defined in the EOL nutrition context, it remains unclear who provides support and assistance or where families can seek information about EOL nutrition care. Nurses are highlighted throughout the literature as a key human resource whose proximity to families and other HCPs leads to naturally occurring opportunities for conversations about care [64,65]. Since nursing care is fundamental for supporting vital nutritional needs when challenges arise with a patient’s natural oral intake of food, nurses hold the unique position of “skilled companion” for patients and families, which predisposes them to opportunities for building relationships and leading multidisciplinary conversations about a patient’s care [65]. Some of the studies indicated that physicians and physician assistants may assume responsibility for facilitating EOL care decisions. Without defined roles, there remains hesitation and challenges in supporting families with EOL decision-making, particularly when family conflicts arise [13,64,66]. Further complicating the situation is that HCPs hold differing perspectives as to the benefits of ANH or how to engage families in decision-making around EOL nutrition [16,59].

Among HCPs, registered dietitians possess the specialized training in nutrition care that should position them as a key member of the multidisciplinary care team who can facilitate EOL nutrition care discussions with families and substitute decision makers or support other members of the care team [67]. Baird-Schwartz [68] argues that dietitians have a strong role on health care teams, helping to facilitate decisions about eating, drinking, and ANH as a component of care. Surprisingly, the role of dietitians was not addressed in the studies included in this review. In fact, the only paper that mentioned dietitians noted that their absence in the study represented a limitation. Dietitians are poised to make a strong contribution to supportive multidisciplinary care teams for residents at EOL.

A key consideration for the implementation of resources to support decision-making is staff availability. For instance, texture-modified therapeutic diets can be beneficial for residents who experience dysphagia, which is common among residents with severe dementia; however, assistance with eating is often required (e.g., supportive hand feeding, prompting, cueing to swallow). On the other hand, resources to support decision-making can outline other potential treatment options, such as the use of special utensils and oral care. Urban-rural differences in staff availability in LTC have also been shown to impact the uptake of decision aids [49]. Findings showed that rural LTC homes with fewer available staff found greater benefit from resources to support decision-making compared to their more highly staffed urban counterparts [49]. This suggests that decision aids helped to alleviate some of the pressure faced by HCPs, or perhaps supported HCPs and families to use their time together more effectively [49].

Though much of the literature captured in this review focused on supporting families and HCPs in decision-making, the authors of the included papers reiterated that the goal is that decisions be made with a resident-focused approach. Some authors illustrated the components of a resident-focused approach to decision-making [68,69]. For instance, Arcand [70] showed that with appropriate information and communication, families and HCPs can reach a consensus (ideally informed by the resident’s values) even when a resident is unable to participate in the decision-making process, allowing for care provision that supports the resident’s goals. Advance care plans or directives are sometimes conceptualized as processes that help residents and families consider potential scenarios, clarify goals of care, and make decisions to support their personal choices. However, advance care plans vary widely, and their use is not consistent, with evidence suggesting that they lack detail specific to eating and drinking at EOL and can be perceived as not relevant to a specific decision because they are not regularly updated [6]. Resources to support decisions about EOL nutrition care could perhaps be useful to supplement or guide interpretation of the information expressed within an advance care plan.

Cultural differences impact the development and uptake of decision aids for EOL nutrition care in LTC [13,34,70,71]. A notable gap in the existing literature was the limited mention of the cultural relevancy of available decision aids. Given the wide variation in cultural beliefs and values about EOL generally and EOL nutrition care specifically [7], recognition of cultural safety within decision aids is imperative.

Similarly, most of the resources to support decision-making did not include residents or family members/substitute decision makers in the development stage. One study, however, engaged residents living with mild dementia in the co-production process [46]. Although the ratio of residents to HCPs included in the study was low, the authors noted that capturing resident perspectives was valuable for informing the overall design of the decision aid. Since a resident’s goals of care are of central importance to any decision aid, including those designed for use by HCPs [72], engaging residents in the coproduction process is necessary for developing decision aids that represent integrated perspectives and lived experiences [46]. Future studies should draw on diverse groups of residents, staff, and families to generate culturally safe decision aids [71]. Additionally, currently available resources focus primarily on residents living with dementia or those who lack decision-making capacity. More inclusive resources would account for resident and family participation in the decision-making process; engaging residents and families is an important step towards cultivating inclusive LTC homes.

### Limitations

Some limitations of our study should be noted. First, our inclusion and exclusion criteria omitted reviews based on the assumption that all relevant studies would be captured in the search and therefore retrieve only duplicate studies. It is possible, however, that by omitting review articles, we may have missed other authors’ interpretations of the findings. Second, our search strategy focused on retrieving evidence-based resources to support decision-making about nutrition at EOL that could be dispersed and implemented in LTC settings. Thus, more resources could exist but not be reflected in this study.

## 5. Conclusions

Decisions about EOL nutrition and hydration can be complex and multifaceted. Multidisciplinary care teams supporting residents and their families during EOL can benefit from resources to support discussion and facilitate decision-making.

## Figures and Tables

**Table 1:** Search terms.

End of Life	Nutrition	Decision-Making Resources	Long-Term Care
Hospice and palliative care nursing Palliative care Palliative medicine Palliative Terminal Dying Death	Nutr * Nutrition therapy * Patient comfort Sustenance Diet * Enteral nutrition Parenteral nutrition Dehydration Beverages Feeding behaviour Nutrition assessment Nutrition policy Comfort feeding Comfort care Compassionate terminal care Feeding behaviour Subsistence Tube feed * Hydrat * Drink Feed * Eat *	Clinical decision-making * Decision-making *	Long term care * Homes for the aged Nursing home * Hospice * Skilled nursing facilities Aged care * Senior care home Convalescent home Special care home Veteran’s home Skilled nursing facility * Health services for the aged

* denotes ‘wildcard’ symbol used in search to find all variations of the base search term

**Table 2:** Overview of Findings.

First Author, Year, Country	Type of Resource	Purpose	Target Audience for Decision Aid	Sample	Design	Goals of Study	Key Findings	Implications for EOL Nutrition Care	Gaps
Arcand, 2009 [43]; CA	An education program on comfort care and advanced dementia, supplemented with a comfort care booklet.	Pilot a palliative care education program for nurses and physicians in nursing homes.	Nursing home staff	N = 48 (bereaved family members)	Intervention	To pilot the impact of an education program on families’ satisfaction with EOL care in nursing homes.	Communication between staff and families increased post-intervention but did not reach statistical significance.	The educational program and booklet triggered more discussion between staff and families and appeared to have facilitated consensus on EOL nutrition decisions.	The small study sample may have contributed to statistical insignificance. Single site intervention. Poor uptake of the comfort care booklet among participants makes it difficult to discern the impact of the resource.
Arcand, 2013 [44]; CA, FR, JPN	A comfort care booklet on palliative care in dementia.	Test the acceptability of a comfort care booklet among nurses in three countries.	Nurses	N = 188 (nurses)	Survey	To test the acceptability of the booklet to nurses.	Quality ratings for each chapter of the booklet varied across countries, with consistently higher ratings in French Canada and lower ratings in Japan. Acceptability was highest in French Canada, high in English Canada, and acceptable for nurses in France and Japan.	The comfort care booklet was intended to inform families about palliative care options in dementia. Cultural adaptations likely improved the acceptability of the booklet to a limited extent in countries other than Canada. The booklet was well accepted and can support nurses in actively informing families about comfort care options.	Small, nonrepresentative sample size. Low response rate in two regions (i.e., <60% in FR and JPN.)
Chang, 2020 [45]; US	A six-page picture-text resource corresponding to a medical vignette about feeding tube placement for dysphagia.	Evaluate the decision-making capacity of persons living with dementia when using visual aids.	Persons with mild and moderate dementia	N = 20 (people living with mild or moderate dementia)	Experimental	To examine decisional capacity using a visual aid.	Participants had significantly better decisional capacity when supported with a visual decision aid.	Decision-making capacity can be improved for people living with dementia with the use of a visual aid in areas of understanding medical information (coughing/choking; lung infection; inadequate nutrition), evaluating and comparing treatment consequences, and relating information to one’s personal situation.	Small sample. Relied on hypothetical medical vignettes. Specific to feeding tube placement decisions, lacks other nutrition-related questions.
Davies, 2021 [46]; UK	An interactive booklet that highlights the progression of dementia, and several aspects of care/decision-making for resident including those around eating and drinking.	Co-produce a decision aid to support family carers of people living with dementia at the EOL.	Families	N = 33 (11 practitioners, 8 family carers, 4 people living with mild dementia)	Qualitative	To develop a process for designing a decision aid for EOL decisions in dementia through a co-production process, which would include the experiences of the resident with dementia.	Eating/drinking was one of the top four issues included in the final version of the decision aid that was developed. The paper summarizes the method used to comprehensively summarize and incorporate the data collected from each of the groups into one interactive decision aid designed for family carers.	Detailed description of the co-production process for designing decision aids to support EOL decision-making; grounded in theory, evidence, and lived experience. Can be useful to inform future interventions and the development of resources to support EOL decision-making.	Though multidisciplinary, not all roles relevant in LTC were highlighted or used in co-production. The resource was not tested; only the process for designing the booklet was documented. Cultural considerations were not addressed.
Eggenberger, 2004 [47]; US	Consensus building resource identifying those involved in decision-making, how the resident arrived at current condition, their prognosis with care options moving forward, and arriving at the best solution rather than focusing on fixing differences between decision makers.	Provide an ethical framework for nurses to help support families in decisions about ANH at EOL.	Nurses	N/A	Theoretical/Narrative	To provide nurses with a process of decision-making through a framework of ethical principles and evidence-based knowledge, which allows the family and nurse to come to a consensus.	Recommend nurses use a consensus-building model for supporting families in making EOL decisions.	The recommended model can aid nurses in best understanding and supporting families in making EOL ANH decisions.	Supports nurse leadership but does not include a multidisciplinary approach to consensus building. Includes theoretical discussion points to consider but lacks specific guidance on the approach or priority of evidence-based information, which can lead to an inability to reach a consensus
van der Steen, 2013 [48]; NL	A comfort care booklet on palliative care in dementia.	Categorize and compare revisions made to translated versions of a comfort care booklet to understand cultural and ethical sensitivities in dementia care resources.	Healthcare providers and family caregivers	N/A (data source: booklet revisions)	Qualitative Content Analysis of Implementation	To translate and adapt the originally Canadian booklet adapted for use in Italy, Japan, and the Netherlands.	Small adaptations concerned rephrasing; larger adaptations concerned additions regarding ANH in dementia. The adapted booklets for each country varied on three themes: patient-family-provider relationships, patient rights and family position, and the typology of treatments and decisions at EOL.	The respective booklets provide a cross-national perspective on palliative EOL care in dementia and particular sensitivities that are useful for shaping palliative dementia care (e.g., local legal and medical standards).	Though focused on patients/families, the local research teams responsible for translating and adapting the booklet did not include people living with dementia or families.
Ersek, 2014 [49]; US	A printed resource was provided to surrogate decision makers about dementia and options about feeding decisions in the intervention group.	Examine the effectiveness of a decision aid for supporting families in relation to staff levels (e.g., strained health human resources.	Surrogate decision makers	N = 256 (surrogate decision makers in 24 LTC homes)	Randomized Control Trial	To determine the effectiveness of intervention based on staffing levels.	With the use of the printed resource, families experienced reduced decisional conflict and increased conversations about EOL nutrition care in facilities with fewer staff (for example, perhaps provide a ratio of staff to residents?).	In homes with fewer staff, the resource helped to facilitate staff-family conversations about EOL nutrition care decisions. The resource can help relay important information to families in LTC homes with fewer nursing staff available to provide basic education or fundamentals about illness trajectory.	A multidisciplinary approach is not mentioned, which could assist with lower staffing levels of nurse practitioners and physician assistants.
Hanson, 2011 [50]; US	An audio or printed resource outlining feeding options in advanced dementia, including educational information and considerations for each.	Test whether a decision aid improves the quality of decision-making for feeding options for surrogate decision makers for nursing home residents living with advanced dementia.	Surrogate decision makers	N = 256 (resident-surrogate decision maker dyads from 24 LTC homes)	RCT	To determine if a decision aid would facilitate decision-making and reduce decisional conflict.	Surrogate decision makers had increased knowledge, lower decisional conflict, and more frequent conversations with providers, ultimately resulting in an increased trend of dysphagia diets, oral assistance feeding, and staff assistance.	With the use of a decision aid, there is likely to be more discussion around the clinical course/care of the resident and higher quality decision-making.	Population sample not representative (e.g., over half European descent and Protestant). Multidisciplinary approach noted?
Loizeau, 2019 [51]; CH	A printed resource on AH that describes administration, benefits, harms, and alternatives; used to help inform decision makers about AH using evidence-based information.	Apply fact boxes as decision support tools to hypothetical scenarios to determine if fact boxes impact comfort with decision-making, knowledge, or preferences for AH in advanced dementia.	Physicians, families	N= 232 (64 physicians, 100 family members of residents living with dementia, and 68 surrogate decision makers)	RCT	Brief, convenient tools for decision-making for a wide variety of target audiences.	Decisional conflict was significantly lower in the fact box intervention at one-month follow-up; knowledge scores were significantly higher. Fact box intervention did not significantly impact decisions to forgo AH.	Fact boxes can be used as both a communication tool and to aid in decision-making. The resources were versatile, making them accessible in any setting, and they are brief reference guides that can be applicable to HCPs or families.	Relied on hypothetical scenarios. Focuses on AH, not AN. Fact boxes were the same for physicians and families; family carers required a relatively high educational background to understand the information.
Riedl, 2020 [52]; GER	An information booklet provided to caregivers regarding general palliative care.	Develop an informative booklet for caregivers of people with advanced dementia on palliative care issues and to investigate family caregiver knowledge and involvement in decisions before and after studying the booklet.	Family caregivers	N = 38 (patient-caregiver dyads)	RCT	To measure the knowledge gain and increase in conversations/involvement regarding medical care and the decisions of family caregivers who received the information booklet.	Caregivers gained knowledge on 6 palliative care topics, including life prolonging measures (e.g., tube feeding). 80% were more involved in decision-making regarding life prolonging measures, including tube feeding. Caregivers lacked knowledge about palliative care and available services (including EOL nutrition, comfort feeding) before reviewing the booklet.	Use of the resource increased caregiver knowledge of palliative care issues, including tube feeding, and increased their participation in decision-making on topics including life prolonging measures (e.g., tube feeding). The study notes that the booklets cannot simply be translated; considerations of legal and cultural aspects, country-specific standards, and practice when adapting guidance on palliative care are recommended.	Dementia specific. The booklet covers a lot of content and therefore might be long/tedious to read. The resource includes sections on tube feeding and thirst and hunger at EOL, but not general eating/drinking at EOL. The research team noted a lack of registered dietitian involvement in development.
Snyder, 2013 [53]; US	A printed resource that provides information regarding eating/drinking interventions in EOL.	Test whether a resource reduces decisional conflict or increases knowledge about feeding options among surrogate decision makers.	Surrogate decision makers	N = 255 (surrogate decision makers in 24 nursing homes)	RCT	To determine if the resource impacted surrogate decision maker knowledge, decisional conflict, and expectations of tube feeding.	Surrogate decision makers had more knowledge and expected fewer benefits from tube feeding following the use of the resource.	This resource can help in educating and addressing myths surrounding the expectations and benefits of tube feeding for those living with EOL dementia.	Most participants were of similar cultural and religious backgrounds.
Holmes, 2010 [54]; UK	A resource that poses ethical questions to consider and helps guide when deciding if AN is in the best interest of the patient.	Overview/script of ethical questions to guide HCPs in supporting EOL ANH decision-making.	Healthcare Providers	N/A	Clinical narrative	To describe the ethical principles providers must consider through a framework of guidance questions when determining the course of nutrition treatment for a patient.	Presents ethical questions for HCPs to consider with patients/families before commencing AN.	Provides a series of questions that can be used to guide HCPs to support ethical decision-making about AN.	Ethical framework questions are framed only in the specific context of ANH. Culture is not referenced in the framework. Fails to mention multidisciplinary perspectives/participation.
Suter, 2008 [55]; US	A framework for HCPs to guide discussions with patients and families about ANH at EOL to address misconceptions about ANH.	Present a review of evidence on the physiological effects of ANH and a framework for discussion about ANH with patients and families.	Healthcare Providers	N/A	Clinical Narrative	To provide evidence-based advantages and disadvantages to ANH, a framework for discussion, and other supportive resources.	Provides a framework for guiding staff-family discussions about ANH. Summarizes a list of credible resources nurses can use to engage families in meaningful discussions about preferences for ANH.	Resources to assist families with advance directives and for engaging families in discussions about ANH. A 6-step framework can help nurses address common misperceptions about ANH and help families cope with feelings of helplessness. The resources emphasize collaboration and education.	The framework of questions does not direct families to multidisciplinary consultations, such as a dysphagia diet with assistance from an RD. URLs for listed resources are unavailable; a lack of a clear layout for framework questions and a printed guideline may deter nurses from using the recommendations.
van der Steen, 2012 [56]; NL	A comfort care booklet on palliative care in dementia.	Evaluate the content, usefulness, and acceptability of a comfort care booklet among families in three countries.	Families	N = 138 (bereaved family members of LTC residents)	Retrospective Cohort	To evaluate the content, format, usefulness, acceptability, and preferred way of obtaining the booklet.	The contents and format of the booklet were generally endorsed, with higher ratings among Canadian and Dutch families than Italian families. The need for and perceived usefulness of the booklet were almost universally positive.	The booklet is suitable for Canadian and Dutch families but requires additional cultural adaptations for use in Italy.	The retrospective study design may have introduced bias (e.g., receptiveness to information when a family’s loved one was still alive). Small, nonrepresentative sample.
van der Steen, 2021 [57]; NL	A question prompt list about palliative and EOL care in dementia.	Evaluate HCP perceptions of the acceptability and usefulness of a question prompt list for palliative care in dementia.	Healthcare providers	*N* = 66 (practitioners)	Mixed methods evaluation	To evaluate the acceptability and usefulness of a question prompt list for helping HCPs provide palliative EOL care for patients with dementia.	Most practitioners found the question prompt list acceptable; the contents were appreciated, with some concern about information overload.	A question prompt list can be a valuable tool for facilitating staff-family conversations about EOL care.	The question prompt list was not assessed by people living with dementia or their families.

End of Life (EOL); Artificial Nutrition and Hydration (ANH); Not Available (N/A); Artificial Nutrition (AN); Artificial Hydration (AH); Randomized Control Trial (RCT); Healthcare Provider (HCP).
